# Pretreatment Inflammation-Based Markers Predict Survival Outcomes in Patients with Early Stage Hepatocellular Carcinoma After Radiofrequency Ablation

**DOI:** 10.1038/s41598-018-34543-z

**Published:** 2018-11-09

**Authors:** Michelle Ong Chu, Chien-Heng Shen, Te-Sheng Chang, Huang-Wei Xu, Chih-Wei Yen, Sheng-Nan Lu, Chao-Hung Hung

**Affiliations:** 1 Division of Hepatogastroenterology, Department of Internal Medicine, Chiayi Chang Gung Memorial Hospital, Chiayi, Taiwan; 2 Division of Hepatogastroenterology, Department of Internal Medicine, Cebu Doctors’ University Hospital, Cebu, Philippines

## Abstract

The prognostic significance of various systemic inflammation‐based markers has been explored in different cancers after surgery. This study aimed to investigate whether these markers could predict outcomes in patients with early-stage hepatocellular carcinoma (HCC) undergoing radiofrequency ablation (RFA). One hundred eighteen patients with newly diagnosed HCC within the Milan criteria receiving RFA as initial therapy were retrospectively enrolled. Pretreatment inflammation-based markers including the neutrophil to lymphocyte ratio (NLR), platelet to lymphocyte ratio (PLR) and prognostic nutritional index (PNI), together with other clinicopathologic parameters were collected. Cumulative overall survival (OS) and recurrence-free survival (RFS) were estimated by the Kaplan-Meier method and by multivariate analysis using Cox proportional hazard model. The 1-, 3-, and 5-year OS rates of patients were 90%, 67%, and 52%, respectively. Kaplan-Meier curves showed that baseline high NLR ≥ 2.5 (*p* = 0.006), low PNI < 40 (*p* = 0.005), history of end-stage renal disease (ESRD) (*p* = 0.005), non-Child-Pugh class A (*p* = 0.001) and elevated alpha-fetoprotein (AFP) ≥ 200 ng/mL (*p* = 0.005) significantly associated with the poor OS, whereas high PLR ≥ 100 did not. By multivariate analysis, high NLR ≥ 2.5 (hazard ratio (HR) 1.94; 95% confidence interval (CI), 1.05–3.59; *p* = 0.034), low PNI < 40 (HR 0.38; 95% CI, 0.20–0.72; *p* = 0.003), ESRD history (HR 3.60; 95% CI, 1.48–8.76; *p* = 0.005) and elevated AFP ≥ 200 ng/mL (HR 4.61; 95% CI, 1.75–12.13; *p* = 0.002) were independent factors. An elevated AFP level of ≥200 ng/mL was the significant factor associated with intrahepatic new RFS by univariate and multivariate analyses. In conclusion, pretreatment NLR and PNI are simple and useful predictors for OS in patients with early-stage HCC after RFA.

## Introduction

Hepatocellular carcinoma (HCC) is the sixth most common cancer worldwide, being fifth in men and ninth in women^1^. It is the fourth most common cause of cancer-related death^2^. Male to female predominance is greater than 2:1 with HCC, and approximately 83% of the estimated 782,000 new HCC cases in 2012 occurred in less developed regions of the world, with Southeast Asia and sub-Saharan Africa being the high-incidence regions, while Southern Europe and North America are the intermediate regions, and Northern Europe and South Central Asia are the low-incidence regions^3^.

In Taiwan, the crude mortality rate of HCC is 30.21 per 100,000 person-years, which is the first and second leading cause of cancer-related mortality in males and females, respectively^4^. Hence, an active surveillance program has been implemented in this country. HCC is diagnosed at an early stage in 70% of patients and is amenable to potential curative treatments, such as surgical resection and liver transplantation. However, only 40% of HCC patients are candidates for resection due to poor liver function reserve secondary to underlying cirrhosis upon diagnosis^5^. Liver transplantation is another option for curative treatment, but it is not often feasible due to shortage of liver donors. Therefore, non-surgical treatments have been introduced. Among these, local ablative therapies have contributed as curative treatments for early stage HCC^6^.

Percutaneous radiofrequency ablation (RFA) is one of the most widely used local ablation therapies for HCC, with an effective complete ablation rate of 85% for solitary tumor <5 cm in diameter or up to 3 tumors with a maximum size of 3 cm^6,7^. Previous randomized controlled trials have demonstrated no significant differences on survival rates between RFA and surgical resection^8,9^.

In the past decade, studies on systemic inflammatory response are considered as enabling markers to cancer pathogenesis, including the proliferation, invasion, recurrence and metastasis of tumors. Valid indicators to predict the prognosis are important in the therapeutic management options of cancer. Published data revealed that these low cost inflammation-based markers including the neutrophil to lymphocyte ratio (NLR), platelet to lymphocyte ratio (PLR) and prognostic nutritional index (PNI) are associated with prognostic significance in patients with HCC^10–13^. The elevated NLR was correlated with worse prognosis in HCC patients treated by RFA^13^, surgical resection^14^, transplantation^15^, and transcatheter arterial chemoembolization (TACE)^16^. A combination of the NLR and the PLR is a useful predictor for recurrence and prognosis in patients with HCC post RFA^17^.Another marker is the PNI, a combination of the serum albumin concentration and total lymphocyte count. This has been extensively introduced in clinical practice for predicting nutritional status and surgical risks of patients undergoing gastrointestinal cancer surgeries^18^.It has also been found that low PNI can be used to identify patients with increased risk of postoperative complications^19^. To the best of our knowledge, there has been no prior study on PNI in HCC patients undergoing RFA. Therefore, the present study will determine the prognostic impact and clinicopathological correlations of these inflammatory indices in patients with early HCC treated with RFA.

## Methods

### Patients

A retrospective analysis of 118 patients with newly diagnosed HCC within the Milan criteria (solitary nodule ≤ 5 cm; ≤3 nodule, none >3 cm; no macrovascular invasion) receiving RFA as initial therapy in Chiayi Chang Gung Memorial Hospital was conducted between January 2013 and August 2015. The diagnosis of HCC was according to the radiological and/or histological criteria as recommended by the American Association for the Study of the Liver Diseases guidelines^20^. Patients with positive history of chronic systemic inflammatory disease, active concomitant infection, uncontrolled diabetes, heart failure, coronary artery diseases, arrhythmia, and other diseases which might affect the inflammation-based markers were excluded.

Standard demographic and clinicopathological data were collected, including the following: age, sex, body mass index (BMI), routine blood count, liver and kidney functions, alpha-fetoprotein(AFP) and tumor characteristics. Clinically significant portal hypertension (CSPH) was defined as (1) a platelet count <100,000/mm^3^ associated with splenomegaly and/or (2) the presence of oesophageal/gastric varices by endoscopy^21^. The blood tests were routinely obtained in all patients before therapy. Pretreatment inflammation-based markers including the NLR, PLR and PNI were computed. The NLR was defined as the absolute neutrophil count divided by the lymphocyte count^10,13^; the PLR was estimated as platelet count divided by lymphocyte count;^17^ and the PNI was calculated as the sum of serum albumin(g/L) and 5 X absolute lymphocyte count (10^9^/L)^22^. The formula of Fib-4 test was [age (years) x aspartate aminotransferase (AST) (U/L)]/[Platelets (10^9/L^) × sqr alanine transaminase (ALT) (U/L)], and the albumin-bilirubin (ALBI) score was defined as −0.085 × (albumin g/L) + 0.66 × log (total bil irubin μmol/L)^23,24^. Written informed consent was obtained from patients prior to treatment. This study was approved by the Research Ethics Committee of Chang Gung Memorial Hospital and was conducted in accordance with the principles of Declaration of Helsinki and the International Conference on Harmonization for Good Clinical Practice.

### RFA procedure

RFA was performed on an in-patient basis using a Cool-tip RFA electrode (Radionics, Burlington, MA, USA) or Viva RF electrode system (STARmed, Korea). Procedures were done percutaneously, under local anesthesia, by qualified hepatologists with the guidance of real-time ultrasonography (US) using a 17-gauge, 2 or 3-cm needle. To avoid a rapid increase in intra-tumoral pressure, RFA was started at a low power (60 W), and the power was increased in increments of 10 watts/min. After each ablation in the tumor, the electrode was stepwise pulled out with ablation from the insertion site under the tip temperature more than 80 °C to reduce the risk of bleeding and tumor seeding. Creation of artificial ascites by intraperitoneal glucose water injection was used for lesions abutting the visceral organ and abdominal wall.

### Follow-up

All patients were followed up in the out-patient clinic. Abdominal triphasic computed tomography (CT) or magnetic resonance imaging (MRI) was performed 4–6 weeks post-treatment. Treatment response to RFA was considered as complete ablation based on the absence of contrast enhancement or abnormal wash-out within or around the ablation zone. Follow-up included the physical examination; blood exams, including the liver function tests and AFP level assessment; and abdominal US every 3–6 months and triphasic CT scan every 6 months. Overall survival (OS) and recurrence-free survival (RFS) of local recurrence, intrahepatic new recurrence and extrahepatic recurrence were assessed. Local recurrence was defined as locally developing HCC besides the ablation lesion or a new one developing in the same segment. The OS time was defined as the time between the termination of RFA and the date of mortality or the last follow-up. The closing date of the study was 28, February, 2018. The RFS time was defined as the time between termination of RFA and the first recording of recurrence or the date of mortality of patients without evidence of disease recurrence. When recurrent tumors were diagnosed, patients received appropriate treatment, including repeated RFA, percutaneous ethanol injection therapy, TACE, surgical resection, liver transplantation, targeted therapy, chemotherapy, radiotherapy or supportive treatment.

### Statistical analyses

The analysis software used was SPSS for Windows version 18 (SPSS Inc., Chicago, IL, USA). Continuous variables were expressed as the mean ± standard deviation (SD) and compared using Student’s *t* test. Categorical data were presented as frequency and were analyzed using the χ^2^ and Fisher’s exact test. Survival curves were estimated using the Kaplan-Meier analyses, and the differences of survival rates between groups were compared using the log-rank test. Univariate analysis was performed to assess significant differences in clinicopathologic characteristics that influenced the OS after RFA. Multivariate analysis was performed using Cox proportional hazards regression model for significant variables identified by univariate analysis. A time-dependent receiver operating characteristic (ROC) curve analysis was used to determine the cut-off values of AFP, NLR, PLR and PNI. All statistical tests were two-sided and differences were considered significant with a *p* < 0.05.

## Results

### Patient characteristics

The study included 118 patients with early stage HCC who had undergone RFA during the study period, for whom complete data were available. The study group consisted of 74 males (63%) and 44 females (37%) with a mean age of 69 ± 10 years (range 32–90). Fifty-one patients (45%) had concomitant type 2 diabetes mellitus. Eight patients (7%) had end-stage renal disease (ESRD) undergoing maintenance hemodialysis. In terms of etiologies, 88 (75%) patients were positive for anti-hepatitis C virus (HCV) antibody and 32 (27%) patients were positive for hepatitis B virus (HBV) surface antigen (HBsAg). Of them, 13 patients with HBV received NA (nucleos(t)ide analogue) therapy before RFA until the last follow-up and the additional 7 patients received NA after RFA. On the other hand, 24 patients with HCV received interferon (IFN) based therapies before RFA, and 26 patients received IFN (n = 7) or direct acting antivirals (DAA) therapy (n = 19) after RFA, of whom 6 had previous IFN treatment failure. The AFP, liver function tests, white blood cell, neutrophil, lymphocyte and platelet counts, as well as NLR, PLR and PNI, of the patients prior to RFA are shown in Table 1.

**Table 1:** Baseline characteristics of the patients.

Factors	Mean ± SD or n [%]
Age (years)	69.4 ± 10.4
Gender, male/female (%)	74 (63)/44 (37)
Body mass index (per kg/m^2^)	25.2 ± 3.8
Diabetes mellitus (%)	51 (43)
End-stage renal disease (%)	8 (7)
Tumor stage I/II (%)	88 (75)/30 (25)
Tumor number, solitary/multiple (%)	84 (72)/34 (28)
Tumor size, ≥3 cm vs <3 cm	26 (22)/92 (78)
HBsAg positive (%)	32 (27)
Anti-HCV positive (%)	88 (75)
Hepatitis B/C/B + C/NBNC	17 (14)/73 (62)/15 (13)/13 (11)
AFP (ng/mL)	121.7 ± 480.7
AST (U/L)	60.2 ± 43.7
ALT (U/L)	57.2 ± 55.5
Child-Pugh grade A/non-A	87 (74)/31 (26)
Albumin (g/dL)	3.6 ± 0.6
Total serum bilirubin (mg/dL)	1.1 ± 0.6
INR	1.1 ± 0.1
WBC (×10^3^/uL)	4.8 ± 1.9
Neutrophil count (%)	58.6 ± 10.8
Lymphocyte count (%)	29.5 ± 9.7
Platelet count (×10^3^/uL)	122.9 ± 73.0
NLR before treatment	2.4 ± 1.4
PLR before treatment	99.2 ± 61.6
PNI before treatment	42.5 ± 7.5

Hepatitis B, positive hepatitis B surface antigen; C, positive antibody to hepatitis C virus; AFP, α-fetoprotein; AST, aspartate aminotransferase; ALT, alanine aminotransferase; INR, international normalized ratio; WBC, white blood cell count; NLR, neutrophil-to-lymphocyte ratio; PLR, platelet-to-lymphocyte ratio; PNI, prognostic nutritional index.

### OS analyses

**Figure 1:**
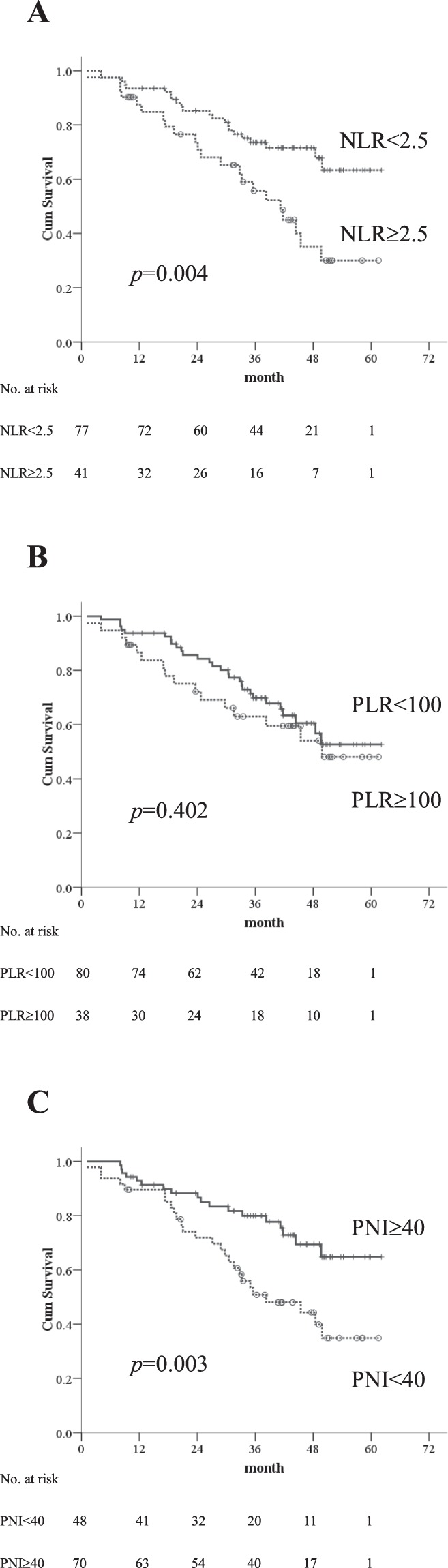
Kaplan-Meier overall survival curves for early stage HCC patients undergoing RFA with low and high inflammatory-based markers (**A**) NLR, (**B**) PLR and (**C**) PNI.

**Figure 2:**
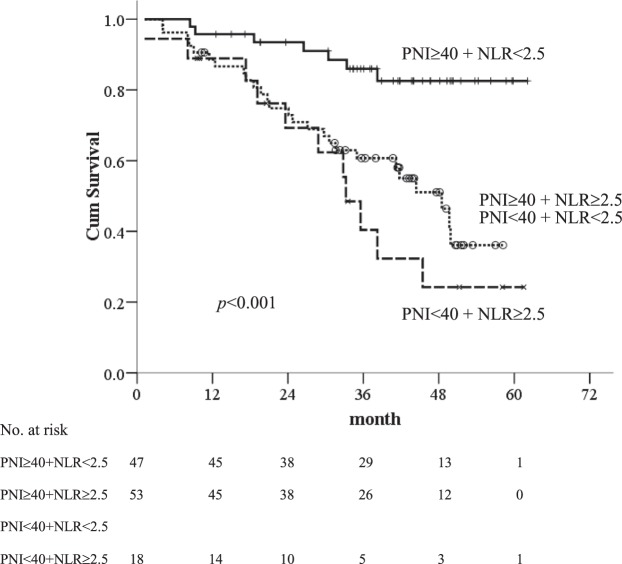
Kaplan-Meier overall survival curves for early stage HCC patients undergoing RFA with combined PNI and NLR.

The median follow-up was 36 months (25–75^th^ percentiles, 20.6–45.2 months). At the time of final analysis, 17 of 118 (14%) patients had died of HCC progression, 12 (10%) was related to liver failure, and 15 (13%) was unrelated to liver disease. Fifty-eight (49%) patients were still alive at their last visit and the remaining 16 (14%) patients were lost to follow-up before the closing date with the median follow-up of 27.5 months (25–75^th^ percentiles, 14.7–38.8 months) after RFA. The 1-, 3-, and 5-year OS rates of patients were 90%, 67%, and 52%, respectively. Based on ROC curves, the cut-off values of NLR, PLR and PNI were 2.5, 100 and 40, respectively. The survival analyses confirmed that the inflammation-based indices were strong predictors of OS, as the median OS durations for patients with low-NLR was 38.1 months (95% CI: 4.1–62.1 months) vs. 33 months (95% CI: 1.2–58.1 months) for those with high-NLR (Fig. 1A). PLR was not a prognostic factor for OS (Fig. 1B). HCC patients with a low-PNI had a median OS of 33.2 months (95% CI: 1.2–58.2 months) vs. 38.2 months (95% CI: 8.1–62.1 months) for those with high-PNI (Fig. 1C). In addition, patients with combined low-NLR and high-PNI had the most favorable outcomes, with a median OS of 38.6 months (95% CI: 8.4–62.1 months) vs. 35.7 months (95% CI: 8.1–52.0 months) and 30.8 months (95% CI: 1.2–58.1 months) for those in the combined high-NLR/high-PNI or low NLR/low PNI and high-NLR/low-PNI groups, respectively. The 1-, 3-, and 5-year OS rates were 96%, 86%, and 83% in patients with combined low-NLR and high-PNI, 89%, 61%, and 36% in those with combined high-NLR/high-PNI or low NLR/low PNI, and 89%, 40%, and 24% in the high-NLR/low-PNI group, respectively (*p* < 0.001) (Fig. 2).

The univariate OS analysis showed significant associations of unfavourable OS with the following parameters: baseline high-NLR (*p* = 0.006), low-PNI (*p* = 0.005), history of ESRD (*p* = 0.005), antiviral therapy after RFA (*p* = 0.003), non-Child-Pugh class A (*p* = 0.001), ascites (*p* = 0.001), ALBI score (*p* = 0.02) and elevated AFP ≥ 200 ng/mL (*p* = 0.005). A high-PLR did not show significance. By multivariate Cox proportional hazards model, high-NLR (HR 1.94; 95% CI, 1.05–3.59; *p* = 0.034), low-PNI (HR 0.38; 95% CI, 0.20–0.72; *p* = 0.003), history of ESRD (HR 3.60; 95% CI, 1.48–8.76; *p* = 0.005) and elevated AFP ≥ 200 ng/mL (HR 4.61; 95% CI, 1.75-12.13; *p* = 0.002) were independent factors for OS (Table 2). In a subgroup analysis of 88 anti-HCV positive patients, multivariate Cox proportional hazards model showed that low-PNI (HR 0.44; 95% CI, 0.22–0.90; *p* = 0.024) and antiviral therapy after RFA (HR 0.05; 95% CI, 0.01–0.39; *p* = 0.004) were significant factors for OS.

**Table 2:** Cox proportional hazards model of baseline prognosticators for overall survival in patients with early stage HCC after radiofrequency ablation.

Factors	Univariate		Multivariate	
	HR (95% CI)	*P*	HR (95% CI)	*P*
Age, per 1 year increase	1.02 (0.98–1.05)	0.323		
Gender, male vs female	1.07 (0.59–1.96)	0.824		
BMI, per kg/m^2^ increase	0.62 (0.34–1.14)	0.126		
Diabetes mellitus, yes or no	1.44 (0.80–2.58)	0.224		
ESRD, yes or no	3.50 (1.47–8.31)	0.005	3.60 (1.48–8.76)	0.005
CSPH, yes or no	2.26 (0.88–5.79)	0.091		
Tumor number, solitary vs multiple	0.84 (0.43–1.64)	0.612		
Tumor stage, I vs II	0.85 (0.43–1.68)	0.646		
Tumor size, ≥3cm vs <3cm	1.79 (0.96–3.32)	0.066		
HBsAg positive, yes or no	1.64 (0.89–3.02)	0.113		
NA before RFA, yes or no*	1.28 (0.47–3.44)	0.631		
NA after RFA, yes or no*	1.20 (0.44–3.33)	0.720		
Anti-HCV positive, yes or no	0.88 (0.46–1.70)	0.710		
IFN before RFA, yes or no**	0.68 (0.29–1.58)	0.368		
IFN or DAA after RFA, yes or no**	0.05 (0.01–0.36)	0.003		
AFP(ng/ml), ≥200 vs <200	3.50 (1.47–8.31)	0.005	4.61 (1.75–12.13)	0.002
Child-Pugh class A vs B or C	0.36 (0.20–0.66)	0.001		
Ascites, yes or no	4.67 (2.48–8.80)	0.001		
NLR ≥2.5 vs <2.5	2.31 (1.28–4.17)	0.006	1.94 (1.05–3.59)	0.034
PLR ≥100 vs <100	1.30 (0.70–2.41)	0.404		
PNI ≥40 vs <40	0.42 (0.23–0.76)	0.005	0.38 (0.20–0.72)	0.003
ALBI grade, 1 vs 2/3	0.42 (0.20–0.87)	0.020		
FIB-4, per 1 increase	1.02 (0.95–1.08)	0.659		

*in 32 HBsAg positive pa lgtients; **in 88 Anti-HCV positive patients.

HR, hazard ratio; CI, confidence interval; BMI, body mass index; ESRD, end-stage renal disease; CSPH, clinically significant portal hypertension; HBsAg, hepatitis B surface antigen; NA, nucleos(t)ide analogue; HCV, hepatitis C virus; IFN, interferon; DAA, direct acting antivirals; AFP, α-fetoprotein; NLR, neutrophil-to-lymphocyte ratio; PLR, platelet-to-lymphocyte ratio; PNI, prognostic nutritional index; ALBI, albumin-bilirubin; Fib-4, Fibrosis-4.

### Recurrence analyses

During the follow-up period, 52, 59 and 24 patients developed local recurrence, intrahepatic new recurrence and extrahepatic recurrence, respectively. The NLR, PLR and PNI did not carry any prognostic significance on the local recurrence, intrahepatic new recurrence and extrahepatic recurrence free survivals (Supplementary Table 1). The 1-, 3-, and 5-year intrahepatic new RFS rates were 74%, 52%, and 43% in patients with combined low-NLR and high-PNI, 67%, 41%, and 38% in those with combined high-NLR/high-PNI or low NLR/low PNI, and 88%, 68%, and 52% in the high-NLR/low-PNI group, respectively (*p* = 0.290).

An elevated AFP level of ≥200 ng/mL was the significant factor associated with intrahepatic new RFS by univariate and multivariate analyses (Table 3). In a subgroup analysis of anti-HCV positive patients, multivariate Cox proportional hazards model showed that antiviral therapy after RFA (HR 0.42; 95% CI, 0.21–0.84; *p* = 0.014) was an independent factor.

**Table 3:** Cox proportional hazards model of baseline prognosticators for intrahepatic new recurrence-free survival in patients with early stage HCC after radiofrequency ablation.

Factors	Univariate		Multivariate	
	HR (95% CI)	*P*	HR (95% CI)	*P*
Age, per 1 year increase	1.00 (0.97–1.02)	0.858		
Gender, male vs female	1.10 (0.66–1.86)	0.709		
BMI, per kg/m^2^ increase	1.17 (0.70–1.94)	0.543		
Diabetes mellitus, yes or no	1.07 (0.64–1.78)	0.786		
ESRD, yes or no	1.24 (0.45–3.44)	0.675		
CSPH, yes or no	1.92 (0.92–3.98)	0.081		
Tumor number, solitary vs multiple	0.72 (0.42–1.24)	0.236		
Tumor stage, I vs II	1.30 (0.75–2.26)	0.352		
Tumor size, ≥ 3 cm vs < 3 cm	1.38 (0.77–2.48)	0.275		
HBsAg positive, yes or no	0.81 (0.45–1.48)	0.502		
NA before RFA, yes or no*	1.62 (0.56–4.62)	0.372		
NA after RFA, yes or no*	1.61 (0.51–5.14)	0.421		
Anti-HCV positive, yes or no	1.09 (0.60–1.98)	0.781		
IFN before RFA, yes or no**	1.01 (0.53–1.92)	0.988		
IFN or DAA after RFA, yes or no**	0.42 (0.21–0.84)	0.014		
AFP(ng/ml), ≥200 vs <200	4.21 (1.71–10.40)	0.002	4.34 (1.75–10.77)	0.002
Child-Pugh class A vs B or C	0.88 (0.49–1.58)	0.662		
Ascites, yes or no	1.10 (0.54–2.24)	0.804		
NLR ≥ 2.5 vs <2.5	1.04 (0.60–1.78)	0.898		
PLR ≥ 100 vs <100	0.69 (0.38–1.24)	0.215		
PNI ≥ 40 vs <40	1.40 (0.60–1.78)	0.231		
ALBI grade, 1 vs 2/3	0.63 (0.36–1.12)	0.116		
FIB-4, per 1 increase	1.02 (0.96–1.08)	0.529		

*in 32 HBsAg positive patients; **in 88 Anti-HCV positive patients.

HR, hazard ratio; CI, confidence interval; BMI, body mass index; ESRD, end-stage renal disease; HBsAg, hepatitis.

B surface antigen; HCV, hepatitis C virus; NA, nucleos(t)ide analogue; HCV, hepatitis C virus; IFN, interferon; DAA, direct acting antivirals; AFP,α-fetoprotein; NLR, neutrophil-to-lymphocyte ratio; PLR, platelet-to-lymphocyte ratio; PNI, prognostic nutritional index; ALBI, albumin-bilirubin; Fib-4, Fibrosis-4.

### Association between the NLR/PNI with various clinicopathological characteristics

The association of inflammatory markers and clinicopathologic features of HCC patients are summarized in Table 4. As previously mentioned that patients were classified into two groups: low-NLR group (65%) and high-NLR group (35%). The high-NLR group were associated with larger tumor size (≥3 cm), lower serum AST level, ascites, higher white blood cell (WBC) count, higher neutrophil and lower lymphocyte counts compared with low-NLR group. There were 70 patients (59%) classified as high-PNI, and 48 patients (41%) as low-PNI. The low-PNI group had significantly worse hepatic function reserve (higher serum AST level, non-Child-Pugh class A and its constitutive variables: ascites, lower serum albumin, higher serum total bilirubin, prolonged prothrombin time and lower platelet count) compared to high-PNI group.

**Table 4:** Comparison of clinicopathologic characteristics between patients with low NLR and high NLR; low PNI and high PNI.

Factors	Low NLR <2.5	High NLR >2.5	*P*	Low PNI <40	High PNI >40	*P*
	n = 77	n = 41		n = 48	n = 70	
Age, years	68.7 ± 10.6	70.7 ± 10.1	0.334	68.4 ± 10.9	70.1 ± 10.1	0.395
Gender, male	47 (61)	27 (66)	0.691	28 (48)	46 (66)	0.44
Diabetes mellitus	28 (36)	23 (56)	0.051	25 (52)	26 (37)	0.131
End-stage renal disease	4 (5)	4 (10)	0.446	4 (8)	4 (6)	0.714
HBsAg positive	20 (26)	12 (29)	0.828	17 (35)	15 (21)	0.139
Anti-HCV positive	60 (78)	28 (68)	0.273	34 (71)	54 (77)	0.52
Major tumor size (≥3 cm)	13 (17)	14 (34)	0.040	10 (21)	17 (24)	0.824
Tumor stage II	23 (30)	7 (17)	0.183	12 (25)	18 (26)	1
AFP (>200 ng/mL)	3 (4)	5 (12)	0.124	3 (6)	5 (7)	1
AST (U/L)	66.6 ± 47.9	48.1 ± 31.5	0.027	73.1 ± 49.3	51.3 ± 37.2	0.007
ALT (U/L)	62.4 ± 56.7	47.5 ± 52.4	0.164	61.0 ± 58.6	54.7 ± 53.6	0.547
Child-Pugh class A	60 (78)	27 (66)	0.189	20 (42)	67(96)	<0.001
Albumin (g/dL)	3.6 ± 0.6	3.6 ± 0.6	0.862	3.0 ± 0.4	4.0 ± 0.4	<0.001
Total serum bilirubin(mg/dL)	1.2 ± 0.6	1.1 ± 0.5	0.456	1.4 ± 0.7	1.0 ± 0.4	<0.001
INR	1.1 ± 0.1	1.1 ± 0.1	0.906	1.2 ± 0.1	1.1 ± 0.1	<0.001
Ascites	9 (12)	12 (29)	0.023	16 (33)	5 (7)	<0.001
WBC (×10^3^/uL)	4.5 ± 1.7	5.3 ± 2.1	0.035	3.9 ± 1.7	5.4 ± 1.7	<0.001
Neutrophil count (%)	52.8 ± 7.9	69.6 ± 5.9	<0.001	58.6 ± 10.8	58.6 ± 11	0.996
Lymphocyte count (%)	34.8 ± 7.3	19.7 ± 4.5	<0.001	29.0 ± 9.8	29.9 ± 9.6	0.606
Platelet count (10^3^/μL)	118.9 ± 58.2	130.5 ± 95.1	0.415	89.7 ± 45.4	145.7 ± 79.6	<0.001

NLR, neutrophil-to-lymphocyte ratio; PNI, prognostic nutritional index; HBsAg, hepatitis B surface antigen; HCV, hepatitis C virus; AFP, α-fetoprotein; AST, aspartate aminotransferase; ALT, alanine aminotransferase; INR, international normalized ratio of prothrombin time; WBC, white blood cell count.

## Discussion

Accumulated evidences have shown strong correlation between inflammation and cancer. A complex tumor microenvironment is one of the most important factors in a cancer prognosis. This has been confirmed that the interaction between the tumor itself and systemic inflammatory response will lead to tumor development^25^. The main features of a tumor-associated inflammatory response are the infiltration of leukocytes, the production of cytokines, the remodelling of tissue and angiogenesis^26^. Particularly in Taiwan, the majority of HCC cases developed from underlying chronic HBV or HCV infections. Both tumor inflammation and immunologic factors are known to enable cancer characteristics, and data support the involvement of both factors in cancer progression and metastasis^26,27^. Therefore, this has led to the development of various inflammatory indices in predicting clinical outcomes of HCC.

Our study showed that high-NLR and low-PNI were independent predictors of OS in early stage HCC patients after RFA. Multivariate analysis also identified other factors, including the serum AFP and ESRD on hemodialysis,which were similar in previous reports^28,29^. The finding of high-NLR concurred with the study of Chen *et al*.^13^ where high baseline NLR was associated with mortality in early HCC after RFA. Although the exact mechanism of high-NLR remains unknown, there are potential accepted explanations for this association. First, increasing neutrophils can promote tumor growth and invasion by releasing the vascular endothelial growth factor (VEGF), series of inflammatory mediators including angiogenesis circulating chemokines (CXCL8), matrix metalloproteinase-8/9 and the anti-apoptotic factor, nuclear factor-κB^30,31^. These inflammatory mediators further lead to oxidative damage, DNA mutation and altered microenvironment, which promote tumor cell growth and progression^32^. Secondly, lymphocytes play a vital role in an adaptive immune system that provide a cellular basis for cancer immunosurveillance and immuno-editing. Therefore, a reduced number of lymphocytes may weaken the ability to inhibit proliferation and metastatic activity of tumor cells despite the presence of cytotoxic cell death and cytokine production^33,34^. Wada *et al*.^35^ reported that increased infiltration of CD4+ T lymphocytes at the tumor margins among HCC patients was associated with a lower recurrence rate and better prognosis. These results suggest that lymphocytes have a role of anti-tumor effect.

The PNI was originally used to assess the immune-nutritional status and surgical risk in patients undergoing gastrointestinal surgery^22^. Subsequently, published data have correlated low-PNI index with tumor development and progression in various gastrointestinal malignancies^19,36,37^. In a meta-analysis by Man *et al*.^38^, preoperative PNI has been shown to be a prognostic predictive factor for OS, disease-free survival and recurrence in patients with HCC. Chan and colleagues^39^ confirmed that a low preoperative PNI was a significant prognostic factor for OS and disease-free survival in patients undergoing curative hepatic resection for HCC. In this study, we provided the first evidence that low PNI predicted OS for early stage HCC patients undergoing RFA. A low-PNI was found to be signficantly associated with poor hepatic function and portal hypertension, which was concordant with the findings reported by Chan *et al*.^39^ who also found poor tumor differentiation, large tumor size, high AFP and old age in addition to poor hepatic function and portal hypertension to be associated with a low-PNI. Valid reasons to explain this association are that low PNI in HCC patients has been associated with an increase in malnutrition, as well as concomitant underlying cirrhosis that might weaken anti-tumor and anti-metastasis response. Furthermore, hypoalbuminemia and lymphopenia have been demonstrated in liver cirrhosis^40^, suggesting that patients with worse hepatic function have lower PNI than those with a better hepatic function.

As to evaluate the prognostic value of combination of NLR and PNI, Okamura *et al*.^41^ presented that both high-NLR and low-PNI were poor predictors of OS in patients undergoing hepatectomy for HCC with curative intent.This was consistent with the findings of the present study which indicated that combined two markers could better reflect the systemic inflammatory response for patients with HCC after RFA, compared with either score alone.

We acknowledged the limitations of our study. First, this was a retrospective cohort in single center. Our predictive markers are needed to be validated in further studies with a multi-center, large sample size and prospective setting. Second, we did not evaluate for other inflammatory factors used in prognostication of HCC, namely C-reactive protein (CRP) and Glasgow Prognostic Score (GPS)^42,43^. The CRP is not routinely taken for examination in the treatment of HCC patients. Further studies are needed to determine which better reflect a poor inflammatory condition or whether combination of markers can improve prognostic performance.

In conclusion, the NLR and PNI serve as an effective inflammatory and immune-nutritional markers in daily clinical practice. Our data confirmed that pretreatment NLR and PNI are simple and useful in predicting the OS of early stage HCC patients undergoing RFA.

## Electronic supplementary material

Supplementary table 1
