# Supplementary material for: Pretreatment Inflammation-Based Markers Predict Survival Outcomes in Patients with Early Stage Hepatocellular Carcinoma After Radiofrequency Ablation
Source: Sci Rep. 2018 Nov 9;8:16611. doi: 10.1038/s41598-018-34543-z (PMC6226503; doi:10.1038/s41598-018-34543-z)
Supplement: Supplementary file 1 — Supplementary table 1 [file 41598_2018_34543_MOESM1_ESM.pdf]

Pretreatment Inflammation-Based Markers Predict Survival Outcomes in Patients with Early Stage Hepatocellular Carcinoma After Radiofrequency Ablation

Michelle Ong Chu[1,2], Chien-Heng Shen[1], Te-Sheng Chang[1], Huang-Wei Xu[1], Chih-Wei Yen[1], Sheng-Nan Lu[1], Chao-Hung Hung[1]

Supplementary TABLE 1 Cox proportional hazards model of inflammation-based markers for recurrence-free survival

	Local recurrence		Intrahepatic new recurrence		Extrahepatic recurrence	
	HR (95% CI)	*P*	HR (95% CI)	*P*	HR (95% CI)	*P*
NLR ≥2.5 vs < 2.5	1.35 (0.77-2.37)	0.288	1.04 (0.60-1.78)	0.898	1.51 (0.66-3.46)	0.331
PLR ≥100 vs <100	1.08 (0.60-1.93)	0.800	0.69 (0.38-1.24)	0.215	1.29 (0.56-2.96)	0.548
PNI ≥40 vs <40	1.07 (0.61-1.87)	0.822	1.40 (0.60-1.78)	0.231	0.80 (0.36-1.81)	0.598

HR, hazard ratio; CI, confidence interval; NLR, neutrophil-to-lymphocyte ratio; PLR, platelet-to-lymphocyte ratio; PNI, prognostic nutritional index
